# Seizure onset zone identification using phase-amplitude coupling and multiple machine learning approaches for interictal electrocorticogram

**DOI:** 10.1007/s11571-022-09915-x

**Published:** 2022-12-07

**Authors:** Yao Miao, Yasushi Iimura, Hidenori Sugano, Kosuke Fukumori, Toshihisa Tanaka

**Affiliations:** 1 Tokyo University of Agriculture and Technology, Tokyo, Japan; 2 Department of Neurosurgery, Juntendo University School of Medicine, Tokyo, Japan; 3 RIKEN Center for Brain Science, Saitama, Japan; 4 RIKEN Center for Advanced Intelligent Project, Tokyo, Japan

**Keywords:** Phase-amplitude coupling (PAC), Electrocorticogram (ECoG), Interictal, Focal cortical dysplasia (FCD), Machine learning

## Abstract

Automatic seizure onset zone (SOZ) localization using interictal electrocorticogram (ECoG) improves the diagnosis and treatment of patients with medically refractory epilepsy. This study aimed to investigate the characteristics of phase-amplitude coupling (PAC) extracted from interictal ECoG and the feasibility of PAC serving as a promising biomarker for SOZ identification. We employed the mean vector length modulation index approach on the 20-s ECoG window to calculate PAC features between low-frequency rhythms (0.5–24 Hz) and high frequency oscillations (HFOs) (80–560 Hz). We used statistical measures to test the significant difference in PAC between the SOZ and non-seizure onset zone (NSOZ). To overcome the drawback of handcraft feature engineering, we established novel machine learning models to learn automatically the characteristics of the obtained PAC features and classify them to identify the SOZ. Besides, to handle imbalanced dataset classification, we introduced novel feature-wise/class-wise re-weighting strategies in conjunction with classifiers. In addition, we proposed a time-series nest cross-validation to provide more accurate and unbiased evaluations for this model. Seven patients with focal cortical dysplasia were included in this study. The experiment results not only showed that a significant coupling at band pairs of slow waves and HFOs exists in the SOZ when compared with the NSOZ, but also indicated the effectiveness of the PAC features and the proposed models in achieving better classification performance .

## Introduction

Epilepsy is a common neurological disease characterized by epileptic seizures that affects 50–70 million people globally (Fisher et al. 2005; Giannakakis et al. 2014; Trinka et al. 2019). Approximately two-thirds of epileptic patients find that their symptoms are adequately controlled by antiepileptic drugs but for the remaining one-third, medication treatment fails (van Mierlo et al. 2014; Miller and Hakimian 2013; Kelly and Chung 2011). For patients who have medically refractory epilepsy, epilepsy resection surgery may be the only possible effective option (Sheng et al. 2018). Given that the aim of epilepsy resection surgery is the resection of the epileptogenic focus, it is crucial to localize as accurately as possible the brain region where the seizures originate, which is termed the seizure onset zone (SOZ), in the pre-surgical evaluation (Martinez-Vargas et al. 2017).

Electroencephalography (EEG) has been considered one of the most important techniques in the diagnosis and treatment of patients with epilepsy during the pre-surgical process (Gibbs et al. 1968; Pillai and Sperling 2010; Avoli 2012; Panteliadis et al. 2017; Yang et al. 2018). In particular, electrocorticogram (ECoG) plays a significant role in the delineation and localization of the SOZ in the pre-surgical treatment owing to its high precision (Tran et al. 1997; Berger et al. 1993). In addition, as a major recording method in the clinical diagnosis of epilepsy, interictal ECoG is widely used for epileptic focus detection (Kuruvilla and Flink 2003; Pilcher et al. 1993). In clinical practice, the SOZ is generally judged by epileptologists visually examining and manually capturing the character of seizures from long-term ECoG recordings. However, the long-term visual inspection of ECoG is time-consuming, laborious, and error-prone (Xanthopoulos et al. 2009; Brenner et al. 2008). Therefore, it is necessary to establish advanced feature extraction and automatic SOZ localization methodologies to provide technical support in the diagnosis and treatment of epilepsy.

The interaction between different frequency bands of brain rhythms has attracted significant attention from researchers, particularly for SOZ localization, over the last ten years. Phase-amplitude coupling (PAC), as a typical form of brain rhythm interaction, has been considered a promising biomarker for SOZ identification in recent investigations. The study conducted by Edakawa et al. (2016) indicated that PAC is more useful to detect the ictal state than the use of high $\gamma$ amplitude alone for patients with temporal lobe epilepsy (TLE). Ibrahim et al. (2013) found that PAC between high frequency oscillations (HFOs) and alpha band is strong in the SOZ when compared with non-epileptic zones. Surveys such as that conducted by Amiri et al. (2016) showed that PAC between high-frequency rhythms (gamma/ripple) and slow waves is more evident in the SOZ compared to that in normal regions for patients with focal epilepsy. In another study, Cámpora et al. (2019) found that higher PAC exists in the SOZ than in the brain region far from Besides, Ma et al. (2021) illustrated that PAC of intracranial EEG during the inter- and pre-seizure stage can provide an accurate reference for epileptogenic zone localization. Liu et al. (2021) found increased theta-gamma PAC in the seizure region at the ictal state. Nevertheless, there is a lack of comprehensive consideration for the PAC of interictal ECoG among wider frequency bands, especially in the fast ripple band, which is one emphasis of our research.

Most importantly, given that the PAC features may vary among different individuals and change with time, it is necessary to conduct automatic feature extraction and classification approaches for SOZ identification. Over the past few years, classical machine learning algorithms and artificial neural networks have played a crucial role in disease diagnosis because of their automatic learning ability from local patterns of inputs (Yu et al. 2020a, b; Abbasi and Goldenholz 2019). Recently, machine learning models have increasingly been applied to identify the SOZ (Islam et al. 2022; Si 2020; An et al. 2020). In particular, some studies have combined PAC feature extraction methods and machine learning approaches to perform SOZ detection. Elahian et al. (2017) suggested that the logistic regression classifier based on PAC between the phase of 4–30 Hz and high gamma amplitude can effectively identify the SOZ. The study from Varatharajah et al. (2018) reported the feasibility to combine PAC and support vector machine (SVM) for automated SOZ identification with an area under the curve (AUC) of 0.73. However, it should be noted that the number of electrodes labeled as SOZ is commonly much lower than the number of electrodes labeled as non-seizure onset zone (NSOZ), which has caused the problem of an imbalanced dataset for SOZ detection and poor classification performance for the classification predictive modeling. Thus, it is a challenge to improve the predictive performance of machine learning models based on the imbalanced dataset for SOZ detection. However, there are few studies on automatic SOZ identification under the imbalanced situation. This is the other issue addressed in our article.

Therefore, in this study, to explore the significant difference of PAC between the SOZ and the NSOZ in HFOs and low frequency bands (0.5–24 Hz), we applied the mean vector length modulation index (MVL-MI) method and statistical measures including mean and Mann-Whitney U test to interictal ECoG recorded from seven patients (adult: four, child: three). Furthermore, to improve the performance of SOZ identification in the imbalanced input case, we established machine learning models, including SVMs, a light gradient boosting machine (LightGBM), and a 2-D convolutional neural network (CNN), introduced the novel feature-wise/class-wise re-weighting technologies, and proposed a time series nest cross-validation (TSNCV) based on PAC features. We formulate the following hypotheses: (1) The PAC between HFOs amplitudes and low frequency phases can serve as a better feature to identify the SOZ when compared with the PAC between ripple amplitudes only and low frequency phases for adult patients. (2) The coupling is not obvious for child patients when compared with adult patients. (3) Our proposed feature-wise/class-wise re-weighting models have a high prediction accuracy for SOZ identification of interictal ECoG in the case of imbalanced dataset situations.

## Materials and methods

**Fig. 1:**
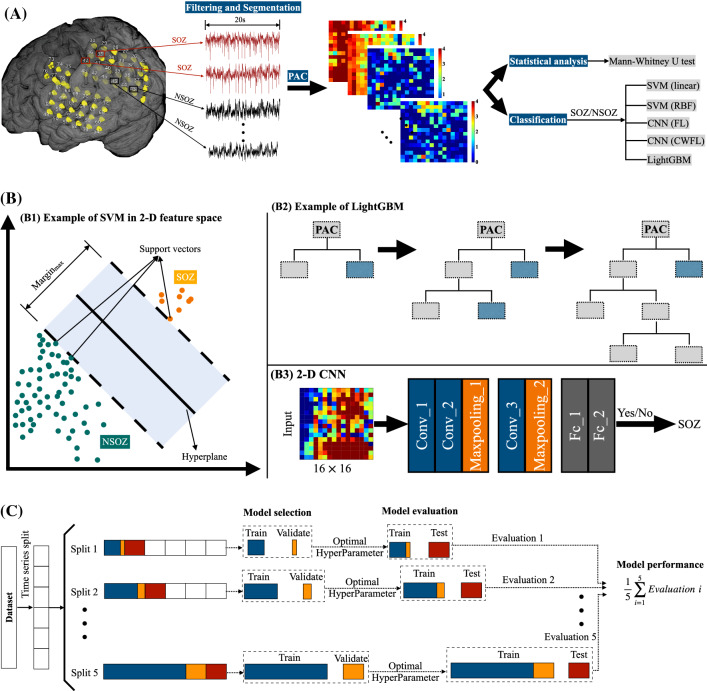
The work scheme, classifiers, and TSNCV principle. **A** The work scheme. **B1** The example of SVM for simple binary classification in 2-D feature space. **B2** The example of leaf-wise tree growth for LightGBM. **B3** The architecture of the established 2-D CNN model. The input is the comodulogram with the size of $16\times 16$. There are three convolutional layers and two fully connected layers named $Conv\_1$, $Conv\_2$, and $Conv\_3$, $Fc\_1$, and $Fc\_2$, respectively. MaxPooling is respectively followed by $Conv\_2$ and $Conv\_3$. **C** The working principle of TSNCV. The dataset is chronologically split into five splits. For each split, there are model selection and model evaluation processes. The training subset, validation set, and test set are denoted by dark blue color, dark orange color, and brown color, respectively, in which the training subset and validation set are selected the former 80$\%$ and the latter 20$\%$ of the training set

As depicted in Fig. 1A, this study consisted of three parts. In the first part, ECoG recorded from patients was analyzed using the PAC approach and was displayed by comodulogram. Second, PAC comodulogram features were analyzed using statistical measures. In the third part, the PAC features were classified between SOZ and NSOZ by employing five models.

### Patients

We included one continuous hour of interictal ECoG data from seven patients (male: 5, female: 2; age: 5-39 years) with focal cortical dysplasia (FCD). All seven patients fell into FCD type 2A/FCD type 2B and had a seizure free outcome after surgery based on the Engel classification system assessment and the follow-up clinic visit evaluation (Wieser et al. 2003). The data were acquired at the hospital from November 2016 to July 2018. The location of electrodes was determined based on a non-invasive diagnostic protocol including seizure semiological evaluation, interictal scalp EEG, magnetic resonance imaging (MRI), fluorodeoxyglucose-positron emission tomography, psychomotor-development testing, and video-EEG monitoring for each patient before surgery (Iimura et al. 2016). The research was approved by the ethics committee at Juntendo University Hospital and the Tokyo University of Agriculture and Technology, and all patients signed consent forms.

### ECoG acquisition

The ECoG signals were recorded using the Neuro Fax digital video EEG system (Nihon Kohden, Inc., Tokyo, Japan) with a sampling frequency of 2,000 Hz. Epileptologists verified the number of electrodes for each patient based on the observations from ECoG recordings. The range of the number of electrodes for the seven patients was 36–76. The SOZ and NSOZ were labeled to the corresponding electrodes according to the judgment of two epileptologists. The information of age, gender, number of electrodes labeled by SOZ, number of electrodes labeled by NSOZ, lesion site, location, pathology, and duration of follow-up for the seven patients is summarized in Table 1 (Akter et al. 2020a, b).

**Table 1:** Summary of information for seven patients

Patient ID	Age	Gender	#SOZ electrodes	#NSOZ electrodes	Lesion Site	Location	Pathology	Duration of follow-up
Pt1	15	M	7	63	$^1$Lt superior parietal lobule	Cortical surface	Type 2B	5 years
Pt2	32	M	10	56	Lt superior parietal lobule	Bottom of sulcus	Type 2B	5 years
Pt3	25	M	16	60	Lt angular gyrus	Bottom of sulcus	Type 2A	5 years
Pt4	39	F	10	40	Lt dorsal superior frontal gyrus	Bottom of sulcus	Type 2B	3 years
Pt5	6	M	3	33	$^2$Rt dorsal middle frontal gyrus	Cortical surface	Type 2B	3.5 years
Pt6	5	F	3	57	Lt dorsal superior temporal gyrus	Cortical surface	Type 2B	3 years
Pt7	5	M	6	36	Lt cingulate gyrus	Bottom of sulcus	Type 2B	3.5 years

$^1$Lt is the abbreviation of left.

$^2$Rt is the abbreviation of right

### Preprocessing

One continuous hour of interictal ECoG data were band-pass filtered by employing finite impulse response with Kaiser window (scipy.signal.firwin function, Python), in which the length of filter was set to odd in order to avoid zero response at the Nyquist frequency (Virtanen and Gommers 2020). In related works (Motoi et al. 2018; Nonoda et al. 2016; Tamrakar et al. 2022), PAC in the narrow band of slow wave (0.5–8 Hz) phase attracted more interest from researchers. In addition, the PAC computation for narrow band pairs was time-consuming and the CNN model design needed to consider the size of the input, in which the input size was related to the frequency bandwidth. Therefore, for each electrode, the ECoG data were filtered between 0.5 and 24 Hz with 0.5/1/2 Hz bandwidth for the low-frequency rhythms and 80–560 Hz with a bandwidth of 30 Hz for the high-frequency components. Specifically, to analyze the coupling of low-frequency signals in detail, especially for the $\delta$ band (0.5–4 Hz) and $\theta$ band (4–7 Hz), different bandwidths were set, in which the bandwidth of frequency band for 0.5–1, 1–8, and 8–24 Hz were set to 0.5, 1, and 2 Hz, respectively. Then, the instantaneous phase of low-frequency filtered data and the instantaneous amplitude of high-frequency filtered signals were calculated using the Hilbert transform method (Feldman 2001). Then, each 1 h-long section of phase/amplitude data was fragmented into 180 segments, each of 20-s duration.

### Calculation of mean vector length modulation index

The PAC value was quantified employing the MVL-MI measure (Canolty et al. 2006). Specifically, the low-frequency phase and high-frequency amplitude were denoted as $A_{amp}(t)$ and $\phi _{pha}(t)$, respectively. The $A_{amp}(t)$ was projected on $\phi _{pha}(t)$ to form to a complex component as $z(t)=A_{amp}(t)e^{i\phi _{pha}(t)}$. The raw modulation index was then defined as $MI_{raw}=\left| \overline{z(t)}\right|$ by calculating the absolute of mean vector of *z*(*t*). From the definition of *z*(*t*), the length of *z*(*t*) was based on the amplitude. Thus, the surrogate data approach was used to z-score the $MI_{raw}$ using the mean and standard deviation of 100 surrogate data, where the mean and standard deviation of surrogate data were denoted as $\mu$ and $\sigma$, respectively. Specifically, the surrogate data was generated by recomputing the complex components 100 times based on the shuffling phase time series, which was generated by adding each time a random time shift, defined as ${z_{surrogate}}_{i}(t)=A_{amp}(t)e^{i{\phi _{pha_{shuffle}}}_{i}(t)}(i=1,\dots ,100)$. ${z_{surrogate}}_{i}(t)$ and ${\phi _{pha_{shuffle}}}_{i}(t)$ indicate the surrogate data and shuffle phase time series, respectively. The final modulation index was defined as $MI=(MI_{raw}-\mu )/\sigma$. It is noted that the surrogate data was generated under the null hypothesis of no modulation by randomly shuffling the phase time series to disrupt the phase-amplitude relationship (Allen et al. 2011; Cohen et al. 2009).

For multiple sub-bands PAC calculation, the phase-amplitude comodulogram was introduced for graphical display of the PAC values over a grid of frequencies (Tort et al. 2010). The horizontal axis was used to represent the frequency of phase and the ordinate axis displayed the frequency of amplitude. The PAC value at the corresponding band pair was denoted by using a pseudocolor plot with the hot color representing a high value.

**Fig. 2:**
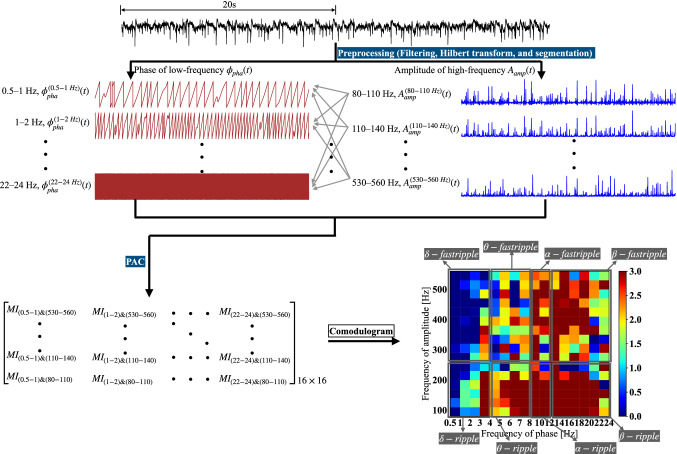
The PAC computation process. ECoG, phase, and amplitude are marked by a black line, brown line, and blue line, respectively. $\phi ^{(0.5-1\;Hz)}_{pha}(t)$ and $A^{(80-110\;Hz)}_{amp}(t)$ denote the phase time series in the band of 0.5–1 Hz and the amplitude time series in the band of 80–110 Hz, respectively. $MI_{(0.5-1)}$ & ${(80-110)}$ is the PAC value between the phase in the band of 0.5–1 Hz and the amplitude in the band of 80–110 Hz. In the comodulogram, the x-axis and y-axis denote the frequency of phase and the frequency of amplitude, respectively. The red color indicates a higher value. The region surrounded by a grey rectangle represents the PAC values in the given band pair

In this study, the PAC values between the phase in the range of 0.5–24 Hz with different bandwidths of 0.5/1/2 Hz and the amplitude of 80–560 Hz with the bandwidth of 30 Hz were computed and displayed for each segment of ECoG data, as shown in Fig. 2. Therefore the number of PAC values for each segment was $16\times 16$. The frequency ranges for the bands of $\delta$, $\theta$, $\alpha$, $\beta$, *ripple*, and *fastripple* were 0.5–4, 4–8, 8–12, 12–24, 80–260, and 260–560 Hz, respectively. There were eight band pairs for PAC, that is, $\delta -ripple$, $\delta -fastripple$, $\theta -ripple$, $\theta -fastripple$, $\alpha -ripple$, $\alpha -fastripple$, $\beta -ripple$, and $\beta -fastripple$, respectively.

### Classifiers

In this study, both classical machine learning algorithms and artificial neural networks were employed for SOZ identification based on PAC features. In detail, five models including SVM with linear kernel, SVM with radial basis function (RBF) kernel, LightGBM, 2-D CNN with focal loss function, and 2-D CNN with class-weighted focal loss function were introduced. To address the issue of imbalanced classification, we applied the class-wise re-weighting approach to SVM and LightGBM to recompute the class weight, and improved the 2-D CNN by introducing a focal loss function, which uses the feature-wise re-weighting measure with re-weighting of the samples. Besides, the input for the 2-D CNN was the comodulogram with a size of $16\times 16$ for each segment, and for SVM and LightGBM, the input was expected to flatten the feature dimension from the 2-D array with a size of $16\times 16$ to a 1-D array with a size of 256. All the models run on iMac Pro (2017) configured with the processor of Intel Xeon W-2191B. We calculated the execution time for each model to train and test each patient’s data and took the average of the execution times of each model. The average execution time of the five models is summarized in Table 2.

#### Support vector machine model

SVM is a common supervised learning model for real-world binary classification, which is based on the statistical learning theory (Vapnik 2010). A simple example of SVM in 2-D feature space is illustrated in Fig. 1B1. The optimal separating hyperplane is defined as that not only classifying the two categories but also ensuring that the margin between the samples from the two categories and the hyperplane segmentation boundary is maximum. Common kernel functions include the linear, sigmoidal, polynomial, and RBF kernels. In this study, SVMs with linear and RBF kernels were employed. There are two typical hyperparameters *C* and *gamma* in SVM, in which the hyperparameter *C* is used to determine the penalty for misclassifying during the fit of the model and the hyperparameter *gamma* is employed to provide a curvature weight of the decision boundary. As the ratio of the two-class samples was imbalanced, as listed in Table 1, the class-wise re-weighting approach was applied to handle the imbalanced classification for the SVM in this study, which consists of weighting the hyperparameter *C* in proportion to the importance of the two classes. Specifically, the weight of the majority class was decreased while the weight of the minority class was increased to prevent misclassification, as expressed in the following formula:

1 $$\begin{aligned} C_i=C\times \omega _{i}, \end{aligned}$$

where *C* is the penalty, $C_i$ is the *C* value of class *i* and $\omega _{i}$ is a weight inversely proportional to the frequency of class *i*. In detail, $\omega _{i}$ is calculated as in Pedregosa et al. (2011):

2 $$\begin{aligned} \omega _{i}=\frac{N_{samples}}{N_{classes}\times {N_{samples_{i}}}}, \end{aligned}$$

where $N_{samples}$, $N_{classes}$, and $N_{samples_{i}}$ are the number of total samples, number of classes, and number of samples that belong to class *i*, respectively.

#### Light gradient boosting machine model

LightGBM is a novel distributed gradient boosting model based on the decision tree algorithm (Ke et al. 2017), as presented in Fig. 1B2. In this study, the LightGBM Python module was used (Microsoft no date). The weight for each class was automatically adjusted by setting the parameter $class\_weight$ to *balanced* mode to handle the imbalanced classification issue. In addition, we also conducted parameter tuning to improve the model performance. These parameters are the number of leaves per tree $num\_leaves$, maximum tree depth $max\_depth$, boosting learning rate $learning\_rate$, and minimal number of data in one leaf $min\_data\_in\_leaf$. Specifically, $num\_leaves$ is one of the key parameters to control the model complexity. A larger value of $num\_leaves$ results in higher accuracy on the training set with a higher risk of overfitting. $max\_depth$ limits the max depth of each tree, and it is commonly set together with $num\_leaves$ to tune the optimal value of $num\_leaves$ and avoid overfitting. $learning\_rate$ is the parameter to control the error corrected speed from each tree to the next. $min\_data\_in\_leaf$ is an essential parameter to avoid overfitting in the training process. We set the values of these four parameters for tuning to [35, 40, 45, 50, 55, 60, 65], [4, 6, 8, 10], [0.01, 0.05, 0.1, 0.15], and [20, 40, 60, 100], respectively.

#### 2-D convolutional neural network model

Each segment of the phase-amplitude comodulogram can be converted into an image with $16\times 16$ pixels. In this study, we proposed a 2-D CNN network structure for focal identification based on the obtained comodulogram results. As described in Fig. 1B3, two convolution blocks are included in the proposed model. The first convolution block consists of two convolutional layers, a batch normalization after each convolutional layer, and a MaxPooling layer. The former and latter convolutional layers have 32 and 64 convolution kernels, respectively. The kernel size was set to $3\times 3$ with the stride of 1 in both the two convolutional layers. The MaxPooling layer, with a size of $2\times 2$, followed. The second convolution block comprised one convolutional layer of 128 kernels with a kernel size of $3\times 3$ and a stride of 1, a batch normalization, and a MaxPooling layer of $2\times 2$. The rectified linear unit (ReLU) activation function and He Uniform initialization were used in all three convolutional layers. Stacked behind the two convolution blocks there were two fully connected layers. The first fully connected layer used the ReLU activation and a dropout rate of 0.5, whereas the second one applied a sigmoid activation. The values of epoch and batch size were set to 100 and 128, respectively. Besides, early stopping was used in this model to prevent overfitting. The proposed model was implemented in Keras 2.3.1 with Tensorflow 1.14.0 backend (Chollet et al. 2015).

#### Focal loss function

As can be observed in Table 1, the comodulogram results used for classification are imbalanced, and the ratio of SOZ to NSOZ samples for patients ranges from 1 : 3.75 to 1 : 19. To address the imbalanced classification, a feature-wise re-weighting approach, the focal loss function, was introduced in this work. The focal loss function is a modulating term of the cross-entropy (CE) loss function that makes the model focus training on hard samples by down-weighting of natural samples, and the definition is as follows (Lin et al. 2017).

Taking the binary classification in the cross entropy loss as an example, the classification loss is the summation of the entropy of each training sample where the weight of each sample is the same. The formula is shown as

3 $$\begin{aligned} CE(p,y)= {\left\{ \begin{array}{ll} -\log (p)&{} \text {if y=1,}\\ -\log (1-p)&{} \text {otherwise,} \end{array}\right. } \end{aligned}$$

where *y* is the label of two classes in the binary classification model with the range of $y\in \left\{ \pm 1\right\}$, and $p\in \left\{ {0,1}\right\}$ is the model predicted probabilities of the samples with label $y=1$. For simplicity, $p_{t}$ is defined as

4 $$\begin{aligned} p_{t}= {\left\{ \begin{array}{ll} p &{} \text {if y=1,}\\ 1-p &{} \text {otherwise,} \end{array}\right. } \end{aligned}$$

and *CE*(*p*, *y*) can be updated to

5 $$\begin{aligned} CE(p,y)=CE(p_{t})=-\log (P_{t}). \end{aligned}$$

To deal with the obstacle that the number of samples for the two classes varies significantly, a common practice is to introduce weights to both the positive and negative samples, down-weighting the negative samples if the negative class frequency is higher, while increasing the weights of positive samples with lower positive class frequency. Thus, the weighting factor $\alpha \in \left\{ {0,1}\right\}$ is introduced to control the shared weights of the two-class samples to the total loss. In a similar way to define $p_{t}$, $\alpha _{t}$ is defined as

6 $$\begin{aligned} \alpha _{t}= {\left\{ \begin{array}{ll} \alpha &{} \text {if y=1,}\\ 1-\alpha &{} \text {otherwise}, \end{array}\right. } \end{aligned}$$

and the balanced cross entropy can be defined as

7 $$\begin{aligned} CE(p_{t})=-\alpha _{t} \log (P_{t}). \end{aligned}$$

Moreover, to control the weights of hard samples and easy samples, a modulating factor $(1-P_{t})^{\gamma }$ is introduced to focus learning on hard samples by reducing the weights of easy samples. Therefore, the $\alpha$-balanced variant of the focal loss is defined as

8 $$\begin{aligned} FL(p_{t})=-\alpha _{t}(1-P_{t})^{\gamma }\log (P_{t}), \end{aligned}$$

where $\gamma$ is focusing parameter with the range of $\gamma \ge 0$. In this study, the ranges of parameters $\alpha$ and $\gamma$ were set to $\left[ 0.75,\;0.9,\;0.99,\;0.999,\;0.9999 \right]$ and $\left[ 0.5,\;1,\;2,\;5 \right]$, respectively.

#### Class-weighted focal loss function

The other loss function we introduced to address the issue of imbalanced input is the class-weighted focal loss function. It improves the performance of the loss function on imbalanced data by introducing a weighting factor that is inversely proportional to the effective number of samples (Cui et al. 2019). Specifically, $N(N_\ge 1)$ is assumed as the volume of data in the feature space of a class. The effective number of samples is defined as

9 $$\begin{aligned} E_{n}=\frac{1-\beta ^{n}}{1-\beta }(\beta \in \left[ 0,1\right) ), \end{aligned}$$

where *n* is the number of samples for the class, and $\beta =\frac{N-1}{N}$. The weighting factor is simply defined as $\frac{1}{E_{n}}$ to balance the loss. Thus, the class-weighted focal loss function is defined as

10 $$\begin{aligned} CB_{focal}(p_{t})=\frac{1}{E_{n}}(1-P_{t})^{\gamma }\log (P_{t}) =\frac{1-\beta }{1-\beta ^{n}}(1-P_{t})^{\gamma }\log (P_{t}), \end{aligned}$$

and for the experiment of the 2-D CNN with class-weighted focal loss function in this study, the range of parameters $\gamma$ and $\beta$ and were set to $\left[ 0.5,\;1,\;2,\;5 \right]$ and $\left[ 0.75,\;0.9,\;0.99,\;0.999,\;0.9999 \right]$, respectively.

### Time series nest cross-validation

To tune the hyperparameters and ensure a robust evaluation of the model, a cross-validation measure was used in this work. In particular, because the comodulogram changes over time, the time series nest cross-validation (CV) based on our classification models was proposed. Moreover, due to the limited comodulogram dataset, the training dataset was split into a training subset and a validation set according to the chronological order to prevent overfitting during model training. As presented in Fig. 1C, the time series nest CV consisted of an inner loop and an outer loop, in which the inner loop was a normal CV with a grid search function for tuning parameters, while the outer loop was used to estimate the performance with the optimal hyperparameters obtained from the inner loop. Specifically, the dataset was chronologically split into $K+1$ folds using the sklearn.model$\_$selection.TimeSeriesSplit function (Pedregosa et al. 2011). In the *K*th split, the K folds were used as the training set, and the $(K+1)$th folds as the test set. *K* was set to five in this work. Besides, the training set was further split into a training subset and a validation set by selecting the former 80$\%$ and the latter 20$\%$ of the training set, respectively. For each inner loop, the model was trained on the training subset and validated on the validation set in conjunction with grid search to select the optimal hyperparameters. Afterward, the model was trained on the training set with the optimal hyperparameters obtained from each inner loop, and the model performance was estimated on the test set. The final estimation of the outer loop was obtained by calculating the average of estimations from all splits. Specifically, the number of splits was set to five in this study. In the *K*th split $(K=1,2,\dots ,5)$, the number of segments for the training subset, validation set, and test set were defined as $N_{train}=K\times {30}\times {N_{electrode}}\times {80\%}$, $N_{validation}=K\times {30}\times {N_{electrode}}\times {20\%}$, and $N_{test}=30\times {N_{electrode}}$, in which $N_{train}$, $N_{validation}$, $N_{test}$, and $N_{electrode}$ indicated the number of training subsets, validation sets, test sets, and electrodes.

### Model evaluation

The metric used in the model evaluation is the AUC of the operating characteristics. Moreover, to obtain the prediction of the probability that each channel of each patient belongs to class SOZ, we took the mean of the probability prediction for each channel of all splits and displayed the mean values intuitively using a horizontal bar chart and a heat map of MRI.

## Results

In this section, we calculated PAC features with one hour of interictal ECoG data recorded from each patient by employing the MVL-MI approach illustrated in "Calculation of mean vector length modulation index" Section. In detail, we computed the PAC values of each segment between the phase of 0.5–24 Hz with various 0.5/1/2 Hz bandwidth and the amplitude of 80–560 Hz with 30 Hz bandwidth, yielding 16 and 16 sub-bands each. The PAC values of each segment at different band pairs were displayed using a comodulogram. We first tested these comodulograms with statistical analysis measures including mean, violin plot, and Mann-Whitney U test. Hereafter, we conducted a classification to identify the SOZ using the established models.

**Fig. 3:**
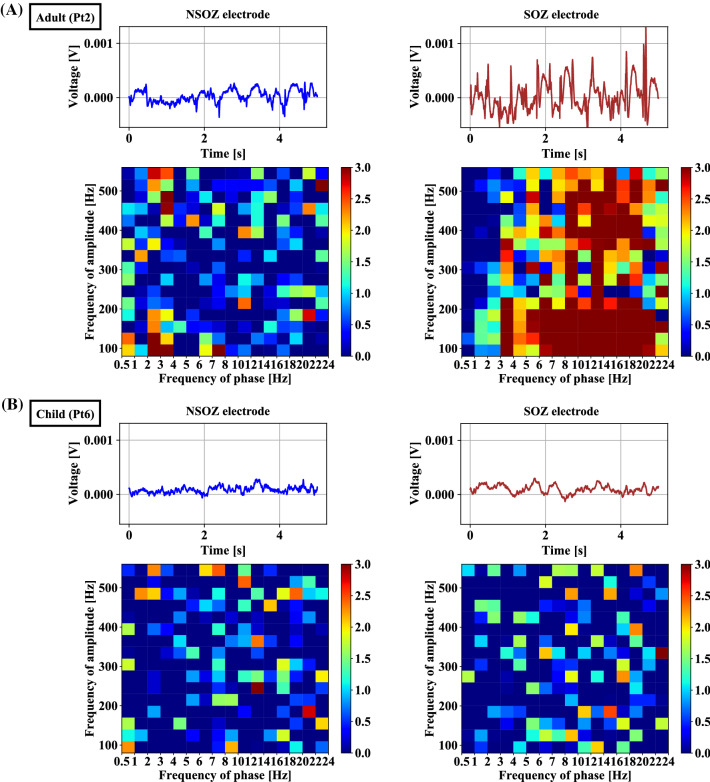
Raw interictal ECoG and comodulogram plots in the SOZ and NSOZ for adult patient Pt2 (sub-figure A) and child patient Pt6 (sub-figure B)

### Comodulogram with respect to raw ECoG

The time-domain in the SOZ (brown) and NSOZ (green), along with the PAC values with comodulograms, are represented in Fig. 3 for both groups (adult vs child). It is observed that a prominent relation exists for the adult patients at the band pairs of $\delta /\theta /\alpha /\beta -HFO$ in the SOZ compared to that in the NSOZ of the child patients. Similar evidence was observed in the SOZ with spikes for the time-domain analysis.

### Statistical analysis with two groups (Adult vs Child)

**Fig. 4:**
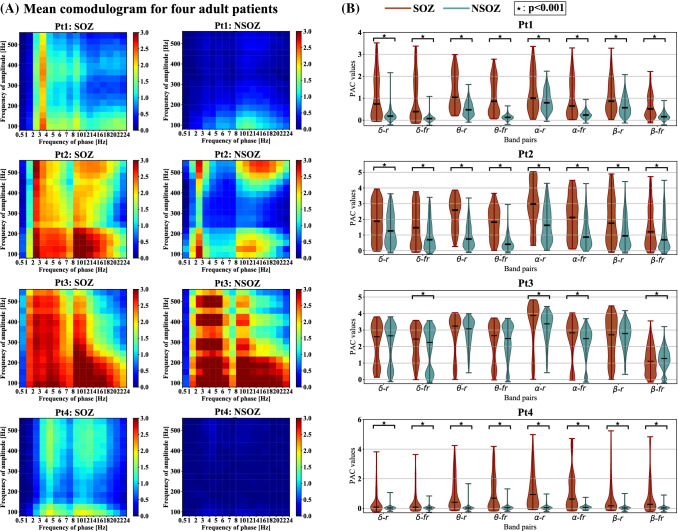
The mean comodulogram and the Mann-Whitney U test results at eight band pairs in both SOZ and NSOZ for four adult patients. **A** The mean comodulogram of SOZ and NSOZ for each adult patient. For each mean comodulogram, the x-axis denotes the frequency of phase, the y-axis indicates the frequency of amplitude, and the pseudocolor represents the PAC value at different band pairs. **B** The Mann-Whitney U test results of the SOZ and NSOZ for four adult patients at eight band pairs. The median value of each band pair for both SOZ and NSOZ is displayed zoom in the most right sub-figure. The eight band pairs are $\delta -r$, $\delta -fr$, $\theta -r$, $\theta -fr$, $\alpha -r$, $\alpha -fr$, $\beta -r$, and $\beta -fr$, representing $\delta -ripple$, $\delta -fastripple$, $\theta -ripple$, $\theta -fastripple$, $\alpha -ripple$, $\alpha -fastripple$, $\beta -ripple$, and $\beta -fastripple$, respectively, where the frequency range of $\delta$, $\theta$, $\alpha$, $\beta$, *ripple*, and *fastripple* is 0.5–4, 4–8, 8–12, 12–24, 80–260, and 260–560 Hz, respectively. The x-axis and y-axis imply types of band pair and PAC values, respectively. The brown and green colors indicate the SOZ and the NSOZ, respectively. $\star$indicates $p<0.001$

**Fig. 5:**
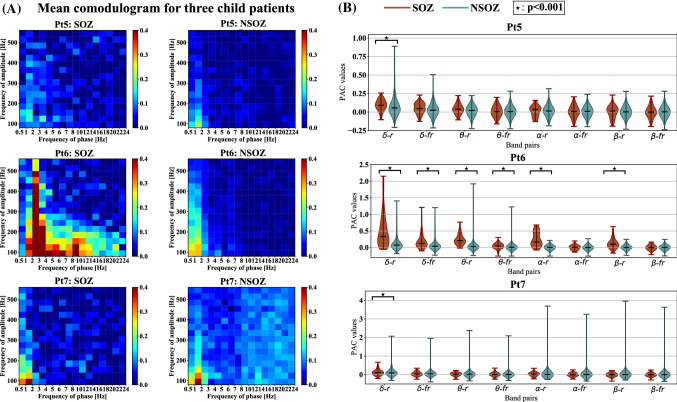
The mean comodulogram and the Mann-Whitney U test results at eight band pairs in both the SOZ and NSOZ for three child patients. **A** The mean comodulogram of the SOZ and NSOZ for each child patient. **B** The Mann-Whitney U test results of SOZ and NSOZ for each child patient at eight band pairs

**Fig. 6:**
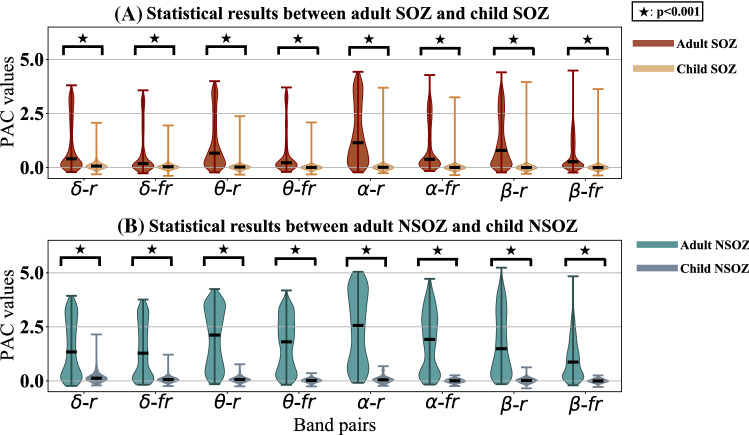
Statistical results between the adult SOZ and child SOZ groups, as well as between the adult NSOZ and child NSOZ groups. The brown, yellow, green, and slate grey colors indicate the adult SOZ, child SOZ, adult NSOZ, and child NSOZ, respectively. $\star$implies $p<0.001$

To observe intuitively the differences in coupling between the SOZ and NSOZ for each patient, we took the mean comodulogram of both the SOZ and NSOZ groups. The number of samples of SOZ and NSOZ for each patient is listed in Table 1. As can be observed in Fig. 4A and Fig. 5A, there exist apparent differences in the mean comodulogram between the SOZ and NSOZ. Besides, it could be visually observed that the coupling for the SOZ mainly happened at band pairs of $\delta /\theta /\alpha /\beta -HFO$ when compared with the NSOZ for four adult patients. Meanwhile, for three child patients, the coupling results are different. Specifically, the differences in coupling between the SOZ and NSOZ groups are weak (Pt5) and even the coupling value for the SOZ is lower when compared to that for the NSOZ (Pt7). There exists obvious coupling in the band pair of $\delta /\theta /\alpha /\beta -ripple$ and $\delta /\theta -fastripple$ for Pt6. Furthermore, the total coupling strength for four adult patients is much stronger than the PAC strength for three child patients.

As illustrated above, the coupling mainly focuses on the band pairs of $\delta /\theta /\alpha /\beta -HFO$. Therefore, the PAC values of each band pair with seven patients were extracted for statistical analysis. Specifically, the PAC comodulogram of each electrode for each patient was averaged along with the time axis. Then PAC values in the averaged PAC comodulogram at the combinations of eight bands ($\delta -ripple$, $\delta -fastripple$, $\theta -ripple$, $\theta -fastripple$, $\alpha -ripple$, $\alpha -fastripple$, $\beta -ripple$, $\beta -fastripple$) of each electrode for each patient were extracted. Every band pair of the PAC value distribution was displayed using the violin plot approach for both the SOZ and NSOZ groups for each patient. The Mann-Whitney U test was introduced to test whether there was a significant difference in the PAC value distribution between the SOZ and NSOZ at the eight designated band pairs at a significance level of 0.001. As shown in Fig. 4B, the median PAC value of the SOZ was higher than that of the NSOZ at the band pair of $\delta -fastripple$, $\theta -ripple$, $\theta -fastripple$, $\alpha -ripple$, and $\alpha -fastripple$ for all four adult patients, which is consistent with the observation in Fig. 4A. Besides, the Mann-Whitney U test results showed that the PAC of the SOZ significantly differed from the PAC of the NSOZ for three adult patients (Pt1, Pt2, and Pt4) at all eight band pairs at a significance level of 0.001. Meanwhile, in the case of child patients (Pt5 and Pt7), this was only significant at the band pair of $\delta -ripple$, as observed in Fig. 5B. The Mann-Whitney U test results between the adult SOZ group (Pt1, Pt2, Pt3, and Pt4) and child SOZ group (Pt5, Pt6, and Pt7), along with the results between the adult NSOZ group (Pt1, Pt2, Pt3, and Pt4) and child NSOZ group (Pt5, Pt6, and Pt7) are summarized in Fig. 6. It can be observed that the coupling of the adult SOZ group and adult NSOZ group is significantly stronger when compared to that of the child SOZ group and child NSOZ group, respectively.

### Results of classification

We used the proposed SVM with linear kernel, SVM with RBF kernel, LightGBM, 2-D CNN with focal loss function, and 2-D CNN with class-weighted focal loss function in conjunction with the proposed TSNCV to classify the SOZ and NSOZ based on the PAC features for the individual patient. The number of splits in TSNCV was set to five. The final result was presented by mean value with standard deviation. The AUC was used to evaluate the model classification performance. Table 2 presents the average execution time for the five models, which were 13061.6, 4201.07, 883.35, 12735.65, and 7500.57 s, respectively. Table 3 summarizes the SOZ prediction results using five classifiers in the band pair of (0.5-24 Hz) &(80-560 Hz) and using three classifiers in the two band pairs, including (0.5-24 Hz) &(80-260 Hz) and (0.5-24 Hz) &(260-560 Hz), for seven patients. It can be observed that the classifiers can better detect the SOZ electrodes of adult patients based on the PAC features, whereas they fail to recognize the SOZ electrodes of children. Besides, when comparing the results of another four models (SVM with linear kernel, LightGBM, 2-D CNN with focal loss function, and 2-D CNN with class-weighted focal loss function), we observed that the results computed by the SVM with RBF kernel were slightly better, with an average AUC for four adult patients of 0.915. In particular, for the AUC of Pt2 using the SVM with RBF kernel, it reached 0.963. Moreover, the AUCs of three adult patients (Pt1, Pt2, and Pt3) were higher than 0.9 when computed by the five classifiers. The average AUC in the band pair of (0.5–24 Hz) &(80–560 Hz) was higher than that in (0.5–24 Hz) &(80–260 Hz).

**Table 2:** Results of average execution time (s) for the five classifiers

Model	$\hbox {SVM}_{{Linear}}$	$\hbox {SVM}_{{RBF}}$	LightGBM	$\hbox {CNN}_{{FL}}$	$\hbox {CNN}_{{CWFL}}$
Time (s)	13061.6	4201.07	885.35	12735.65	7500.57

**Table 3:** Results of AUC for seven patients using five classifiers

Band range	Method	Adult Pt1	Adult Pt2	Adult Pt3	Adult Pt4	Child Pt5	Child Pt6	Child Pt7	Mean of Adult	Mean of Child	Mean of all
(0.5–24 Hz) & (80–560 Hz)	$^1$ $\hbox {SVM}_{{Linear}}$	0.909±0.014	0.940±0.001	0.900±0.023	0.847±0.013	0.489±0.026	0.780±0.171	0.501±0.034	0.899±0.033	0.590±0.134	0.767±0.178
	$^2$ $\hbox {SVM}_{{RBF}}$	0.912±0.014	0.963±0.011	0.938±0.020	0.847±0.015	0.493±0.027	0.772±0.195	0.492±0.024	**0.915±0.043**	0.586±0.132	0.774±0.187
	LightGBM	0.904±0.012	0.957±0.005	0.935±0.010	0.837±0.017	0.505±0.028	0.747±0.154	0.494±0.045	0.908±0.045	0.582±0.117	0.768±0.182
	$^3$ $\hbox {CNN}_{{FL}}$	0.922±0.007	0.935±0.024	0.931±0.007	0.819±0.020	0.508±0.027	0.732±0.145	0.493±0.029	0.902±0.048	0.578±0.109	0.763±0.179
	$^4$ $\hbox {CNN}_{{CWFL}}$	0.909±0.034	0.935±0.031	0.902±0.048	0.824±0.018	0.509±0.024	0.639±0.168	0.503±0.029	0.893±0.041	0.550±0.063	0.746±0.177
(0.5–24 Hz)& (80–260 Hz)	$\hbox {SVM}_{{Linear}}$	0.885±0.024	0.900±0.006	0.878±0.027	0.760±0.128	0.497±0.037	0.780±0.197	0.490±0.050	0.856±0.056	0.589±0.135	0.741±0.164
	$\hbox {SVM}_{{RBF}}$	0.894±0.019	0.952±0.010	0.911±0.018	0.835±0.013	0.504±0.035	0.659±0.297	0.470±0.034	0.898±0.042	0.544±0.082	0.746±0.186
	LightGBM	0.889±0.021	0.935±0.011	0.912±0.021	0.825±0.013	0.502±0.031	0.784±0.107	0.536±0.017	0.890±0.041	0.607±0.126	0.769±0.165
(0.5–24 Hz)& (260–560 Hz)	$\hbox {SVM}_{{Linear}}$	0.889±0.019	0.923±0.004	0.856±0.024	0.722±0.144	0.498±0.026	0.679±0.125	0.499±0.025	0.848±0.076	0.559±0.085	0.724±0.164
	$\hbox {SVM}_{{RBF}}$	0.888±0.018	0.954±0.010	0.906±0.019	0.684±0.241	0.506±0.025	0.643±0.170	0.511±0.023	0.858±0.103	0.553±0.063	0.727±0.175
	LightGBM	0.886±0.016	0.945±0.006	0.901±0.009	0.793±0.007	0.489±0.026	0.648±0.086	0.495±0.022	0.881±0.055	0.544±0.074	0.737±0.179

$^1$ $\hbox {SVM}_{{Linear}}$ is the SVM with the linear kernel.

$^2$ $\hbox {SVM}_{{RBF}}$ is the SVM with the RBF kernel.

$^3$ $\hbox {CNN}_{{FL}}$ is the CNN with the focal loss function.

$^4$ $\hbox {CNN}_{{CWFL}}$ is the CNN with the class-weighted focal loss function

The highest mean AUC value is indicated in bold

**Fig. 7:**
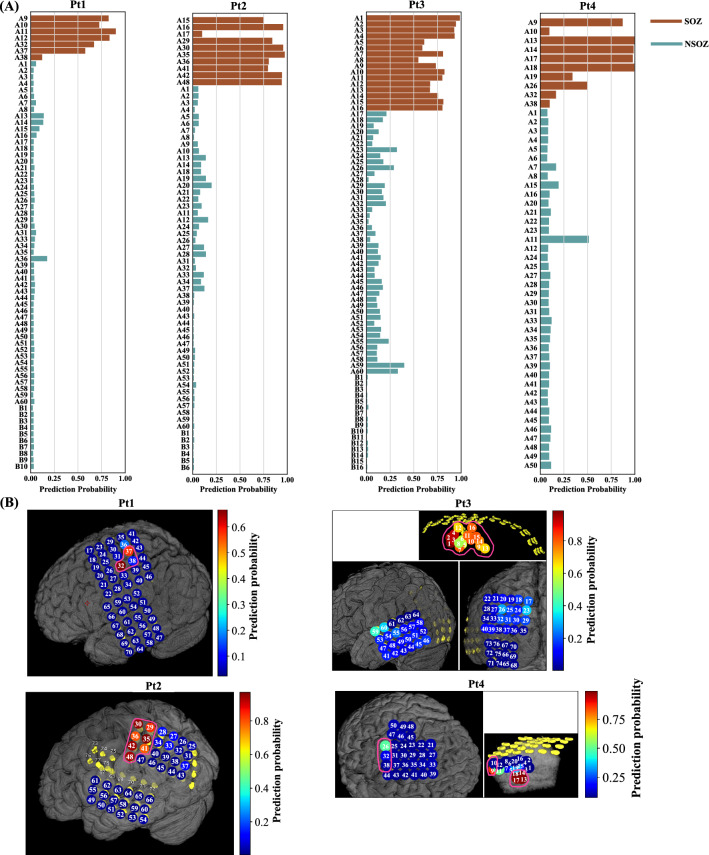
The prediction probability in bar-plot and MRI for four adult patients. **A** The bar plot of the prediction probability of each electrode for four adult patients. The x-axis and y-axis denote the value of prediction probability and channel name, respectively. The brown color indicates the SOZ electrodes, while green represents the NSOZ electrodes. **B** The MRI of prediction probability for four adult patients. The pseudo color denotes the value of the prediction probability of each electrode. It is noted that the numbers 1–60 and 61–76 in (**B**) represent A1–A60 and B1–B16 in (**A**), respectively. Electrodes surrounded by purple lines denote SOZ electrodes

Furthermore, we calculated the mean probability prediction of five splits for each corresponding electrode for four adult patients to demonstrate the probability that the electrode was classified to be a SOZ based on the SVM with RBF kernel. Fig. 7A demonstrates that the probability prediction for SOZ electrodes was high, six out of seven for electrodes A9–A12, A32, and A37. This was much higher than for the NSOZ electrodes of Pt1. Likewise, for Pt2, Pt3, and Pt4, the probability predictions of nine (A15, A16, A29, A30, A35, A36, A41, A42, A48) out of ten SOZ electrodes (A15–A17, A29, A30, A35, A36, A41, A42, A48), 16 (A1–A16) out of 16 SOZ electrodes (A1–A16), and five (A9, A13, A14, A17, A18) out of ten SOZ electrodes (A9, A10, A13, A14, A17–A19, A26, A32, A38) were higher than those of the corresponding NSOZ electrodes, respectively. The location information of electrodes and corresponding values of prediction probability were shown in Fig. 7B. It should be noted that the location information of electrodes A1–A16 for Pt1 and electrodes A1-A24 for Pt2 is not shown in the MRI because these electrodes were placed in the cortical sulcus. In detail, for Pt1, the NSOZ electrode A36 with higher probability prediction was located near the SOZ electrode A37. In addition, the NSOZ electrode A11 with higher probability prediction was located next to the SOZ electrode A9 for Pt4.

## Discussion and conclusion

Recent studies have hypothesized that PAC can reflect information transfer within neurons, especially can strengthen the synchronization between high-frequency components and specific phases of slow wave (Canolty and Knight 2010; Amiri et al. 2016; Allen et al. 2011). We analyzed the coupling characteristics of one hour of interictal ECoG data recorded from seven epileptic patients by employing the MVL-MI method. According to numerous reports, HFO is a promising biomarker of the epileptogenic zone (EZ) (Frauscher et al. 2017; Thomschewski et al. 2019; Park and Hong 2019). However, there is almost no research on the PAC between fast ripple and slow rhythms for SOZ localization. Therefore, we calculated the PAC between the low-frequency phase and the amplitude of HFOs (ripple and fast ripple) for interictal ECoG. The statistical results shown in Fig. 4 verified our hypothesis that there exists significant coupling in the SOZ at band pairs of slow rhythms and HFO when compared with the NSOZ for adult patients. The classification results presented in Table 3 further emphasize the feasibility to detect the SOZ by using the PAC features between slow waves and HFOs. These might indicate the hyperpolarization of cortical neurons due to low-frequency waves co-occurring with interictal HFOs (Steriade and Amzica 1999). In addition, the results of the mean comodulogram also displayed differences in specific coupling band pairs due to individual differences. Besides, a related study reported that the higher PAC values of $\beta -\text {high}~\gamma$ in the ictal state were located around or within the SOZ (Edakawa et al. 2016). Combined with our study, this implies that higher PAC values in both the interictal and ictal states may be used as a promising biomarker to identify the SOZ. These findings motivated the PAC study in the identification of the SOZ.

Moreover, we observed the differences in PAC values between four adult epileptic patients and three child epileptic patients presented in Fig. 4, Fig. 5, and Fig. 6. The range of PAC and the band pairs in which coupling occurs for adult patients are different from those for child patients. Compared with the PAC results for child patients, the range of PAC for adult patients is much higher. This implies that coupling properties may be related to the age of the subject. Furthermore, the PAC between the SOZ and NSOZ for child patients presents no significance in some band pairs. Fig. 3 indicates that the stronger coupling may be correlated to spikes in the time domain. These observations suggest that PAC may be a candidate biomarker to localize the SOZ for adult patients who have prominent coupling, and it is necessary to combine another feature to confirm the position of the SOZ for child patients whose seizures are more complicated compared with those of adult patients.

Because of the time-varying PAC features and individual differences, manual feature extraction was difficult. Therefore, in this study, the classical machine learning algorithms SVM and lightGBM were employed to identify the SOZ automatically. In addition, each segment of PAC at different band pairs displayed by the comodulogram can be considered as an image with $16\times 16$ pixels. Therefore, we also designed the 2-D CNN model to learn and identify the characteristics of inputs automatically. Moreover, as can be observed in Table 1, the number of SOZ samples was substantially larger than the number of NSOZ samples for each patient, resulting in an imbalanced classification situation. To improve the SOZ identification accuracy due to the imbalanced input, we innovatively introduced the feature-wise/class-wise re-weighting technology. Specifically, in SVM and LightGBM, the hyperparameter *C* was weighted to handle the imbalance of classes, in which *C* was in proportion to the importance of the classes. Meanwhile, in the CNN, we consequently introduced two types of loss function (focal loss function and class-weighted focal loss function) to overcome the imbalanced classification. Both loss functions had a good performance for imbalanced inputs. Furthermore, we also proposed the TSNCV for the model to improve the accuracy and unbiasedness of the evaluation of classification performance.

The classification results presented in Table 3 showed that the results obtained using the SVM with RBF kernel were slightly better compared with those obtained using other four models (SVM with linear kernel, LightGBM, 2-D CNN with focal loss function, and 2-D CNN with class-weighted focal loss function) for SOZ identification, with an average AUC for four adult patients of 0.915. From the comparison of execution time and AUC for the five models presented in Tables 2 and  3, the SVM with RBF kernel was the most accurate, whereas the LightGBM was the fastest of all. The LightGBM performs nearly as well as The SVM with RBF kernel. From the comparison of the band range, The AUC in (0.5–24 Hz) &(80–260 Hz) was higher when compared with that in (0.5–24 Hz) &(260–560 Hz), which implied a higher PAC feature importance in the ripple band when compared to the fast ripple. That is, the PAC of ripple activity had a larger effect on the model for SOZ identification. Last but not least, as indicated in Fig. 7B, some NSOZ electrodes with higher probability prediction may be related to the fact that their implant positions were located close to the SOZ region.

This study had three major limitations that could be addressed in future work. First, the research was limited by the small number of patients. With limited subjects, the results might not be generalizable. In future research, we will consider more patients’ data. Another limitation of this research was related to the singleness of features. As indicated in Table 3, the SOZ of adult patients could be predicted accurately with a higher AUC using PAC alone, while it is not easy to identify the SOZ by applying PAC only for child patients. Future research could improve these results by combining PAC with other promising features in child patients. Finally, the research findings of this work were limited by the generality of the proposed model. The proposed model could supply better classification evaluation for certain individuals, whereas it could have a lack of applicability to new patients. Hence, to overcome the drawback of patient-specific models, effective methodologies are required, such as transferring learning, in future research (Desai et al. 2019; LeCun et al. 2015).

Different PAC estimation measures have been used to estimate the coupling characteristics of the SOZ for various categories of epilepsy such as TLE and FCD as illustrated in "Introduction" Section (Edakawa et al. 2016; Amiri et al. 2016; Cámpora et al. 2019; Ma et al. 2021). In this study, two main processes were applied to identify the SOZ, including PAC feature computation and PAC-based classification using machine learning methods. The approach of the PAC comodulogram has a wide frequency range and high frequency resolution by using a narrow bandwidth. The classification is a supervised learning process based on the correct label (SOZ and NSOZ) obtained from doctors. This classification process is data-driven and adaptive to an imbalanced issue. Therefore, the proposed methods could be used as a verification method for the possibility of a label being correct or incorrect in SOZ identification of different categories of epilepsy. However, for clinical practical use, a well-established system including an ECoG viewer and ECoG analysis function using advanced AI approaches would be warranted. This technology can be incorporated into the ECoG viewer and other commercial software.

SOZ localization in the process of drug-resistant epilepsy patient diagnosis using interictal ECoG is challenging. In this study, we applied a PAC calculation approach called MVL-MI to analyze the PAC between slow waves and HFOs and proposed novel models for automatic feature learning and SOZ prediction under an imbalanced situation. The main contributions of this study can be briefly summarized as follows: (1) We comprehensively analyzed the PAC features not only between ripple and slow waves but also between fast ripple and slow waves. (2) We observed the difference in PAC between adult patients and child patients. (3) Given the imbalance between the number of SOZ and NSOZ data, we re-weighted the hyperparameters in SVMs and LightGBM, and introduced the focal loss function and class-weighted focal loss function in the proposed 2-D CNN model, respectively. (4) Because PAC features may vary and change over time, we designed a TSNCV for the prediction of SOZ more accurately and unbiasedly. The final results support our hypotheses presented in "Introduction" Section.
